# *Schistosoma haematobium* detection in snails by DraI PCR and Sh110/Sm-Sl PCR: further evidence of the interruption of schistosomiasis transmission in Morocco

**DOI:** 10.1186/1756-3305-7-288

**Published:** 2014-06-24

**Authors:** Fatima Amarir, Faiza Sebti, Ibrahim Abbasi, Abderrahim Sadak, Hajiba Fellah, Haddou Nhammi, Btissam Ameur, Abderrahman Laamrani El Idrissi, Mohamed Rhajaoui

**Affiliations:** 1 Laboratory of Parasitology, Department of Parasitology, National Institute of Hygiene, Agdal, Rabat, Morocco; 2 Department of Biology, Faculty of Science and Technology, Al Quds University, Jerusalem, Palestine; 3 Department of Biology, University of Mohammed V, Agdal, Rabat, Morocco; 4 Direction of Epidemiology and Control of Diseases, Ministry of Health, Rabat, Morocco

**Keywords:** Schistosomiasis, Elimination, *Bulinus truncatus*, *Schistosoma haematobium*, DraI PCR, Sh110/Sm-Sl PCR, Morocco

## Abstract

**Background:**

This is the first study in Morocco to estimate snail infection rates at the last historic transmission sites of schistosomiasis, known to be free from new infection among humans since 2004. Screening of large numbers of snails for infection is one way to confirm that *Schistosoma haematobium* transmission has stopped and does not resurge.

**Methods:**

A total of 2703 *Bulinus truncatus* snails were collected from 24 snail habitats in five provinces of Morocco: Errachidia, El Kelaa des Sraghna, Tata, Beni Mellal, and Chtouka Ait Baha. All visible snails were collected with a scoop net or by hand. We used waders and gloves as simple precautions. Snails were morphologically identified according to Moroccan Health Ministry guide of schistosomiasis (1982).

All snails were analyzed in pools by molecular tool, using primers from the newly identified repeated DNA sequence, termed DraI, in the *S. haematobium* group. To distinguish *S. bovis* and *S. haematobium*, the snails were analyzed by Sh110/Sm-Sl PCR that was specific of *S. haematobium.*

**Results:**

The results showed that snails from Errachidia, Chtouka Ait Baha, sector of Agoujgal in Tata and sector of Mbarkiya in El kelaa des Sraghna were negative for DraI PCR; but, snails from remaining snail habitats of El Kelaa des Sraghna, Tata and Beni Mellal were positive. This led to suggest the presence of circulating schistosome species (*S. haematobium, S. bovis* or others) within these positive snail habitats. Subsequently, confirmation with *S. haematobium* species specific molecular assay, Sh110/Sm-Sl PCR, showed that none of the collected snails were infected by *S. haematobium* in all historic endemic areas.

**Conclusion:**

The absence of *S. haematobium* infection in snails supports the argument of *S. haematobium* transmission interruption in Morocco.

## Background

In Morocco, urinary schistosomiasis caused by *Schistosoma haematobium*, was first diagnosed in 1914 in Marrakech, and was severely endemic until 1981. Schistosomiasis control has been given a high priority in public health work since 1982 [1]. A four pronged control strategy was employed consisting of (i) annual screening and treatment of human cases, including increased intensity of case detection in health centers and by mobile teams; (ii) malacological control by means of mollusciciding and environmental management (depending on the ecological setting) as a collaborative effort between health, agriculture, forestry, and water resource sectors; (iii) health education and community mobilisation; and (iv) intersectoral collaboration with involvement of services from administration, health, agriculture and education, at local and provincial levels [2].

Great achievements have been seen during the last three decades. The number of schistosomiasis patients dropped from 35596 cases in 1983 to 95 cases in 2003 [3,4]. The regular control efforts and the rainfall deficit (>20%) during 1990–2000 that resulted in the natural drying of irrigation canals decreased densities and snail habitats of *Bulinus truncatus*, a snail which acts as the intermediate host of *S. haematobium*, from 14 provinces with positive *B. truncatus* habitats in 1983 to 7 provinces in 2006 [3-5]. By 2004, the likely interruption of *S. haematobium* transmission had been achieved at national level. Five years after, the process of confirming the interruption of transmission and possible certification of elimination was started with a national serological survey of human schistosomiasis *haematobium* among children born during the elimination phase of the control program. The results showed absence of positive cases [6].

To complement the human testing, study of cercarial shedding by snails is the most useful approach to estimate human to snail transmission. However, cercarial shedding can be highly focal and of low frequency, even in areas of significant transmission [7]. Indeed, considering that prepatent infection can last for several weeks with only a proportion of snails reaching the stage of cercarial shedding, that mortality of infected snails can be higher after cercarial shedding, and that in cold seasons sporocyst development is delayed, it can be assumed that prepatent infection rates can be substantial, and will variably exceed patent infection rates over time [8]. Detection of schistosomal antigens in snail hemolymph is a better predictor of prevalence than cercarial shedding; it is suitable for large-scale screening and detection and more sensitive. However, detection of antigens actively secreted by developing parasites obviously cannot be used as a suitable marker for early infection, but more as an indicator of prepatent and patent infections [9].

In the other hand, those assays cannot discriminate *S. haematobium* from other parasites, especially *S. bovis* that may co-exist in naturally infected snails and may pose problems for the differential diagnosis based on morphology. Molecular tool, such as DraI PCR, had been used in coastal Kenya. The assay was practical for large scale monitoring of *S. haematobium* transmission in affected communities, cheaper and the detection limit was less than 10 fg of *Schistosoma* DNA [10,8,11]. The DraI sequence, not found in *S. mansoni* and *S. japonicum*, and present in DNA of other schistosomes belonging to the *S. haematobium* group [8], represent 15% of the *S. haematobium* genome. Because of the abundance of the sequences, the PCR of DraI sequence could detect very low amount of DNA.

To differentiate the member within the group, PCR of Sh110/Sm-Sl is important. DNA from *S. bovis* was not amplified at all, while *S. intercalatum* and *S. curassoni* could be differentiated from S*. haematobium* by the different banding pattern of their amplification products. The Sh110/Sm-Sl PCR products constitute 0.002% of the genome. This PCR can detect 1 pg of *S. haematobium,* and it is very sensitive and specific [10]. Furthermore, the PCR assay is characterized by lower dependence on participation by local population, amenable to automation and high-throughput testing [11,12].

## Methods

### Study area

**Figure 1:**
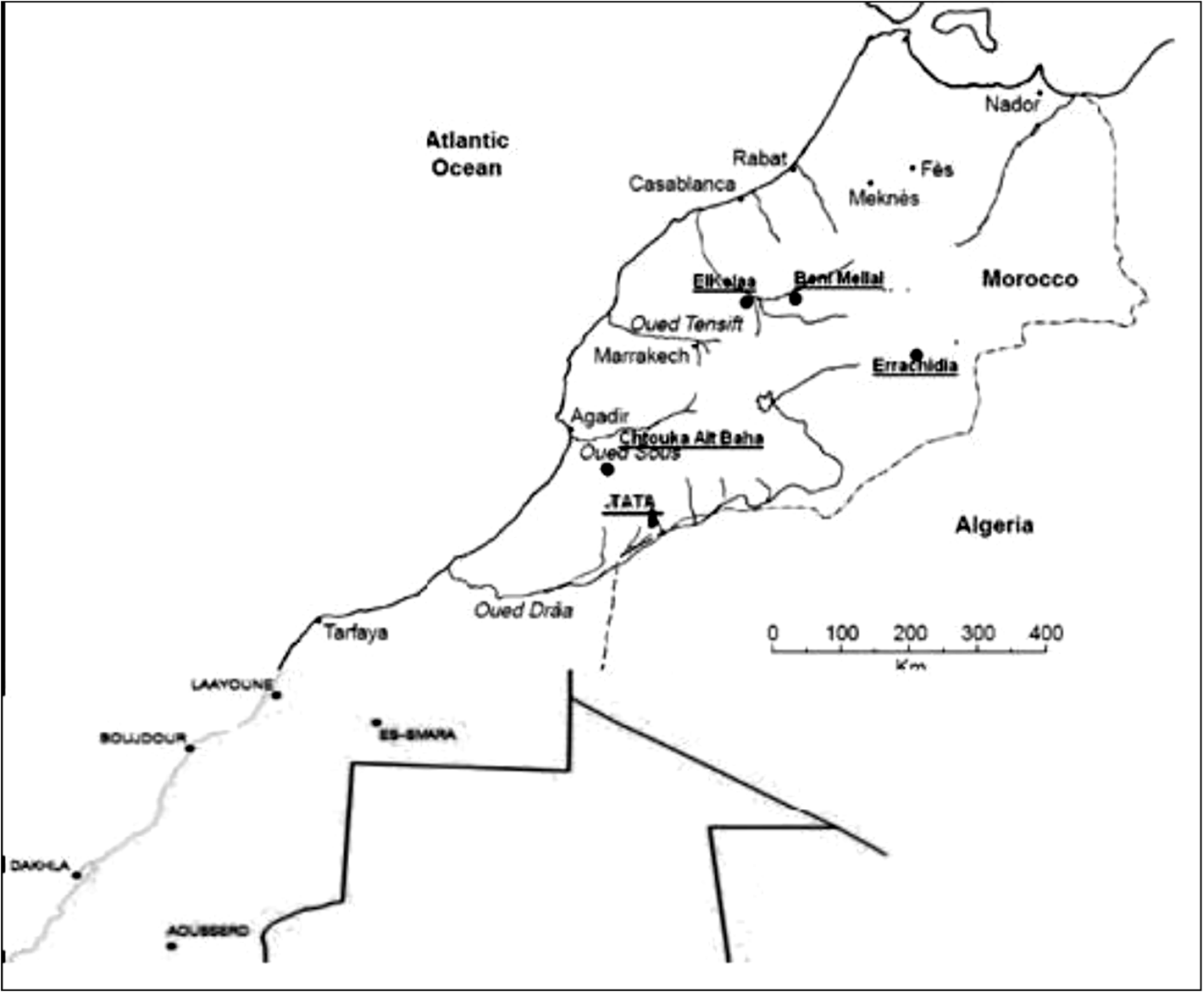
Moroccan map showing selected areas in the study and principal rivers.

We chose the provinces with histories of recent transmission of schistosomiasis, and presence of snail intermediate hosts habitats: Chtouka Ait Baha, Errachidia, El Kelaa des Sraghna, Tata, and Beni Mellal (Figure 1). In each province, we selected sectors and localities where the last known cases were detected. Chtouka Ait Baha has a semi arid climate. The sector of Targa NTouchka was chosen because it represented a hot spot of urinary schistosomiasis and where the last cases were detected in 2003 [13]. Errachidia has a Saharan climate. Locality of Meski, selected in this study, is situated on Oued Ziz, origin of eighty percent of cases in the past. The last case was reported in 2004 [14]. Tata has a Saharan climate. The first serological study, conducted in 2001, showed that sector of Rahala was the hot spot of schistosomiasis [15]. Then we selected this sector and two other sectors in the proximity. El Kelaa des Sraghna province has a semi arid and arid climate. It is a site with history of high incidence and where the last case was detected in 2000 [16]. Beni Mellal has a continental climate. Urinary schistosomiasis was highly endemic in the irrigated agriculture areas (sector of Kourifat and sector of Bouaker). The last two cases were detected in 2000 [16].

### Snail collection

In April 2008, investigations were carried out in any suspected positive site within the chosen sectors. Snails were collected each morning for one week in each snail habitat and they were identified *in situ* by experienced staff. Each 5 to 10 snails, were directly placed in ethanol (70%), and then sent to the laboratory of Parasitology, National Institute of Hygiene at Rabat for morphological examination, identification to species level, and molecular tests for *Schistosoma* parasite identification.

To accurately pinpoint all snails presents in each snail habitat, despite of the continuous chemical and physical treatment, all technicians have chosen the hot season (between April and July) in their respective province, when a probable rapid increase in snail density associated with an increase in temperature was generally observed [17].

### PCR detection of *S. haematobium* in snails

Extraction of DNA: The hexadeclytrimethylammonium bromide (CTAB) method was used as previously described [10,18]. Briefly, three to five snails based on their sizes (estimated to 50 mg, and equal, constant number of snails per group may be better), were crushed and the soft tissue of snails was incubated in lysis buffer (100 mM Tris -HCl pH 7.5, 20 mM EDTA, 1.4 M NaCl, 2% CTAB, and 0.2% 2-Mercaptoethanol) with 500 μl proteinase K (10 mg/ml), at 60°C for 1 to 2 hours until complete digestion. If we use constant number of snails, proteinase K must be added according to the total mass of snail. This was followed by phenol/chloroform DNA extraction and by ethanol precipitation using 3 M sodium acetate and cold ethanol. The precipitated DNA was washed with ethanol 70%, dried, and dissolved in 50 μl of TE buffer.

Amplification by DraI PCR: DraI PCR was carried out in a volume containing 200 μM dNTPs, 25 pMol of each DraI primers (forward: GATCTCACCTATCAGACG, reverse: GTCACCAATAATATGAAAC), 2.5 units of Taq DNA polymerase, and 5 μl of target DNA (not diluted). The final volume for PCR reaction was 50 μl. All PCR assays were carried out by initial denaturation at 95°C for 5 min, 35 cycles of 95°C for 1 min, 72°C for 1 min, followed by final elongation at 72°C for 10 min, and then held at 4°C. If the result of DraI PCR that detects *Schistosoma* DNA was positive, we amplified Sh110-SmSl that was specific for *S. haematobium*.

Amplification by Sh110/Sm-Sl PCR: We used a primer that is targeting a novel repeat sequence of *S. haematobium*: the 525 base pair Sh110 repeat (Sh110: 5’- TTC CTC CAA CTA CCA TCT TAT CTC-3’), and a second primer that was derived from the *S. mansoni* splice leader sequence Sm-SL (Sm-Sl: 5’- AAC CGT CAC GGT TTT ACT CTT GTG-3’). These primers were employed in PCR assays for species-specific discrimination between *S. haematobium* DNA and DNA of other related animal schistosome species. The method used for amplification was identical to that of DraI PCR.

For detecting S. *haematobium*-infected snails by PCR, preliminary experiments were carried out for establishing the optimal conditions for DNA extraction and PCR. Electrophoresis: The amplified DNA products were resolved by agarose gel electrophoresis and stained with ethidium bromide for visual detection by UV trans-illumination. If the band revealed on gel, was bright, indicating high concentration of PCR product, we diluted DNA of 1/10 in PBS.

Positive and negative controls: In each PCR assay, 10 ng of *S. haematobium* purified DNA was used as a positive control and negative control was the water.

## Results

### Abundance of snails

Snail habitats of Errachidia, El Kelaa des Sraghna, Tata, Beni Mellal and Chtouka Ait Baha varied significantly over time by observation of the number of *B. truncatus* snails. Chtouka Ait Baha represented a hot spot of *B. truncatus with* 960 snails. In contrast, snail habitats of Errachidia were the poorest one (101 snails). A total of 2703 snails were collected from all snail habitats, and they are divided into 651 groups of snails.

### Results of DraI PCR

**Figure 2:**
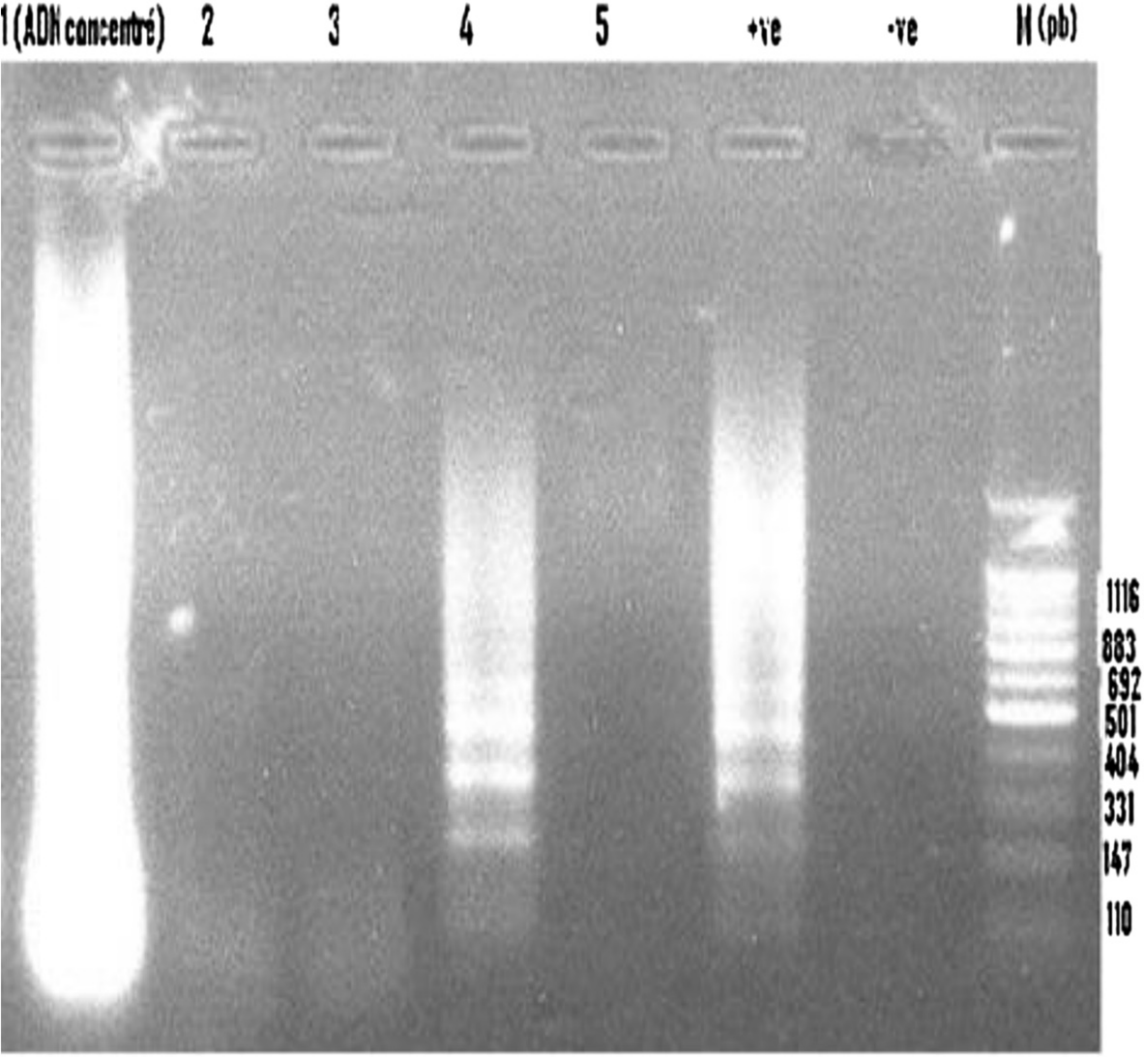
**Agarose gel electrophoresis analysis of DraI PCR amplified products from different collected snails.** Lane 1–5: (pool of 5 snails was tested per lane; Lane 1 and 4: Positive snails; Lane 2,3,5: Negative snails, Lane + ve: Positive control; Lane ve-: Negative control, and M: size marker (base pair).

The molecular identification of snails for infection by *S. haematobium* gave varying results among the province and snails habitat within the same province. Molecular identification of *Schistosoma spp* by DraI PCR showed that none of the snails collected in Errachidia and Chtouka Ait Baha provinces were infected. Also, in Agoujgal locality and one snail habitat (named Mbarkia) in sector of Ouled Marrek, respectively in Tata and El kelaa des Sraghna provinces, none of the snails were infected (Table 1 and Figure 2).

**Table 1:** DraI and Sh110/Sm-Sl PCR results of different collected snails in different sectors and localities

**Province**	**Locality**	**Snails habitats**	**Number of snails collected (number of screening group of 3 to 5 snails)**	**Number of positive snail goups For DraI (and %)**	**Number of positive snail goups for Sh110/SmSl (and %)**
Chtouka Ait Baha	Targa N’Touchka	Aghou Niguer	200 (52)	0	-
		Takchirane/Tizghine	200 (48)	0	-
		Timintoudroute	250 (66)	0	-
		Doussaghouch	210 (54)	0	-
		Tamda N’Benmouss	100 (24)	0	-
Chtouka Ait Baha total		5	960 (224)	0	-
Errachidia	Meski Oued Ziz	Agmmatine and Rjel	12 (4)	0	-
		Louidi	30 (7)	0	-
		Seguia Meski	59 (13)	0	-
Errachidia total		3	101 (24)	0	-
Tata	Agoujgal (2)	Agoujgal/tagoujgalt	150 (34)	0	-
		Agoujgal/toufssirt	150 (36)	0	-
	Rahala (3)	Rahal/ain tahafit	40 (12)	6 (3)	0
		Rahal/ain imazighen	60 (18)	10 (5)	0
		Rahala/ain issoukine	50 (14)	5 (2.5)	0
	Taourirt (2)	Taourirt/ain tichoute	100 (32)	22 (11)	0
		Taourirt/ain tihirite	50 (12)	12 (6)	0
Tata total		7	600 (158)	55 (27.6)	0
El Kelaa des Sraghna	Ouled Marrek	Mbarkia	1O7 (34)	0 (0)	-
		Noujania	3 (1)	1 (0.5)	0
	Coop Falah	D7	77 (17)	9 (4.5)	0
	Coop Chabab	D6	142 (38)	20 (10)	0
	Coop Hahadia	D3	83 (41)	2 (1)	0
		OGG2	20 (4)	2 (1)	0
El Kelaa des Sraghna total		6	432 (99)	34 (17.08)	0
Beni Mellal	Laasara	SD7 10	160 (32)	25 (12.57)	0
		SD6	150 (30)	24 (12.06)	0
	Oulad Abdoun-krifate	D4	300 (84)	61 (30.65)	0
Beni Mellal total		3	610 (146)	110 (55.3)	0
Grand total		24	2703 (651)	199 (100)	0

"-": We dont analyze DraI PCR negative snails.

### Results of Sh110 SmSl PCR

**Figure 3:**
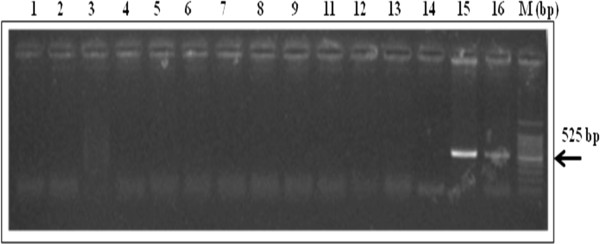
**Agarose gel electrophoresis analysis of Sh110/Sm-Sl PCR amplified products from different collected snails.** Lane 1–11: pool of 5 snails was tested per lane; Lane 12: Negative control; Lane 13 and 14: DNA preparation negative control; Lane 15: Spiking control (*S. haematobium* DNA was added to known negative snail tissue), Lane 16: Positive control (pure *S. haematobium* DNA), and. M: size marker (base pair).

Snails collected from Beni Mellal province and remaining localities of El Kelaa and Tata province were positive by DraI PCR. That suggested the presence of circulating schistosome species (*S. haematobium, S bovis* or others). Confirmation by Sh110/Sm-Sl PCR showed none of DraI PCR positive collected snails were infected by *S. haematobium* (Figure 3).

## Discussion

When prevalence has dropped to a very low level, or is presumed to be zero, looking for infection in snails is probably the best way to monitor transmission, confirm its absence, and monitoring re-emergence of schistosomiasis. Molecular mass screening of pools of snails for schistosomal infection enables coverage of large areas. It is particularly important for periodical monitoring where the prevalence of snail infection is very low and only a few infected snails can be found among thousands of uninfected ones (particularly during the maintenance phase after effective control, or in new water development schemes where the danger of the spread of schistosomiasis exists). The use of this approach in developing countries, requires further studies to be applied to a variety of conditions and to prove the cost effectiveness of the method [19].

In our study, one test PCR test costs about 7 USD; by pooling the DNA into 651 groups of 2703 snails has reduced to 76% the total cost than if we analyze snail by snail.

DraI PCR allows detection of infected snails one hour after snail exposure to miracidia. Thus, it identifies the entire population of infected snails, regardless of whether they eventually shed cercariae, representing in quantitative terms the direct outcome of human contamination of water bodies [8]. This approach has been applied to large-scale monitoring of infection in field snails at transmission sites in Coast Province, Kenya [10]. The sensitivity of this assay was demonstrated by detection of 10 fg of *S. haematobium* DNA [8,10,11]. *Dra*I was not found in *S. mansoni* or *S. japonicum* by dot hybridization. However, because the *Dra*I repeat is present in DNA of other schistosomes belonging to the *S. haematobium* group [18], the identification of *S. haematobium–*infected snails by a simple PCR assay has been limited, thus far, to areas where these other *S. haematobium–*related schistosome species are absent or very rare [8]. Negative results by DraI PCR of snails collected from Errachidia and Chtouka Ait Baha confirm the absence of *S. haematobium* human to snail transmission. However, positive results by DraI PCR, of snails collected from Tata, El Kelaa des Sraghna, and Beni Mellal, suggest *S. haematobium* infection of snails or cross-amplification assays with DNA from a variety of animal schistosomes [19]. *S. bovis* is the most widespread and prevalent species [20,21], and thus has the widest potential geographical overlap with *S. haematobium*. The other animal schistosomes’ species are less widely distributed*. S. mattheei* is found in southern Africa [20], *S. margrebowiei* is found in a relatively small area in west and southern Central Africa [20], *S. curassoni* is found primarily in western Africa [20,22], and *S. intercalatum,* belong to the same group and causing sporadic schistosomiasis in humans, is found in foci in central Africa within Equatorial Guinea, Sao Tome, Nigeria, and Mali [23].

Consequently, DraI PCR cannot discriminate human and animal foci of schistosomiasis in those three areas and thus required to analyse the positive snails by the second Sh110/ Sm-Sl PCR. The negative result of Sh110/Sm-Sl PCR but, DraI PCR positive result could be due to the cross reaction to *S. bovis*; El Kelaa des Sraghna, Tata and Beni Mellal contain the national’s largest ovine and bovine populations.

These approaches will allow extension of large-scale PCR monitoring for snails infected with *S. haematobium*[10], which had been previously limited to areas where cross-reacting (*Dra*I containing) animal schistosome species were absent or very rare. However, the use of two PCR may take more time and products. A drawback of using Sh110 SmSl PCR alone or DraI PCR alone, in a co-endemic area is that they would not be able to detect mixed populations of *S. haematobium* and *S. bovis*. Recently, Webster *et al*., present a high-throughput one-step multiplex COX1 PCR diagnostic method to detect and discriminate between *S. haematobium* and *S. bovis,* and enables the discrimination between false negatives and failed Sh110SmSl PCRs (used alone). The detection sensitivity of COX1 PCR is 0.8 ng of genomic DNA [24]. However, the estimated DNA concentration of a single miracidium is about 1–2 ng/ml, and taking into consideration that less than a tenth of the extracted DNA is used for amplification, and that assay was not tested for direct identification of infected snails, the COX1-based assay, was further refined for use in real-time PCR, with higher detection sensitivity (pg range) [25,26]. More recently, Abassi *et al*. develop a simple and more sensitive PCR assay that enables direct discrimination of *S. haematobium* from related animal schistosomes, by the primer combination of DraI reverse primer and Sh73 direct primer (73d). The sensitivity of *S. haematobium* detection was 1 pg, whereas *S. bovis* detection was 10 pg. It can be assumed that detection of snails infected with *S. haematobium* will be accomplished from very early prepatency. Such assays required further validation using larger numbers of field snails for large scale monitoring of post-intervention residual transmission [25].

Our approache using two simultaneous PCR, DraI PCR and Sh110 SmSl PCR, allowed a good sensitive detection of snails infected with *S. haematobium* from very early prepatency (1 pg) in the maintenance elimination phase of urinary schistosmiasis in Morocco [27]. Also, PCR has been shown to be practical for large-scale monitoring of *S. haematobium* transmission in affected communities [10], collection of snails is simple and can be done by village workers [28], the simple CTAB DNA extraction procedure greatly eliminate the inhibitors, and establishment of the PCR approach for monitoring infected snails with low cost should be developed. Extraction of collected snails in EDTA was shown as suitable for long-term preservation of the DNA at ambient temperature (Hamburger J and others, unpublished data), as has been previously shown with blood and sputum [19]. Although DraI PCR and Sh110 SmSl were highly sensitive and specific for *S. haematobium* detection, research of novel primers that can differentiate human and animal schistosomes simultaneously will greatly decrease the cost of the assays [7]. The use of Loop-Mediated Isothermal Amplification (LAMP) will decrease the cost further down and more field friendly. Studies of feasibility of using LAMP for field studies should be developed [27].

The fact that all snails from the five historic endemic provinces were negative for *S. haematobium* infection, absence of autochthonous parasite since 2004, absence of circulating parasite among children age school demonstrated by the serological study in 2009 [6], support the absence of *haematobium* focus (human and snails) whatever the epidemiological statut of *S. bovis* (animal specie), and that may ensure that transmission does not re-establish

*Planorbarius metidjensis*, experimental intermediate host of *S. haematobium* in south of Morocco, living at high altitude, from 340 m to 1380 m [29], was not collected. However, the fact that, the parasite was not autochthonous in all historic endemic areas since 7 years, and the natural intermediate host of *S. haematobium* in Morocco, *B. truncatus*, was uninfected, led to the assumption that the life cycle of *S. haematobium* is interrupted whatever the intermediate host is.

Here, we caution that some factors – human movement between Morocco and endemic countries, low sensitivity/specificity of current diagnostic tools, changes in environmental and socio-economic factors- may pose obstacles towards the mantain of elimination, and prevention of reemergence of the parasite [30]. Health education should be given to those who will go to the endemic areas to improve their knowledge and awareness on prevention and control of *haematobium* schistosomiasis, there by reducing the risk of exposure to the infested freshwater [31]. Serological survey, and the developpement of highly variable DNA markers, to investigate the genetic epidemiology of *S. haematobium* within both definitive mammalian and intermediate snail hosts is greatly suitable in elimination phase of schistosomiasis in Morocco [32].

## Conclusion

We have shown that molecular tools using pooled snail extracts to testing large sample of snails is feasible and allow to monitor reemergence of schistosomiasis in Morocco. Combination of serological [6] and present malacological surveys reinforce the arguments of interruption of schistosomiasis transmission in Morocco. However, serious challenges for the control program exist due to the limitation of control of immigration from endemic countries and limitation of scientific technology [33].

## Competing interests

The authors declare that they have no competing interests.

## Authors’ contributions

The study was designed by RM and LA. The database of collected snails was communicated by NH, HF and AB. The PCR analysis of snails and interpretation of the results was carried out by A.F and SF. Our PCR results interpretation was revised by AI. AF prepared the draft article. RM, SF, SH, and A.I provided source documents and contributed comments on the draft article. All authors gave final approval of the version for publication. RM coordinated the project with WHO and is the guarantor of the article.
